# Positron Emission Tomography Imaging of Tumor Cell Metabolism and Application to Therapy Response Monitoring

**DOI:** 10.3389/fonc.2016.00044

**Published:** 2016-02-29

**Authors:** Amarnath Challapalli, Eric O. Aboagye

**Affiliations:** ^1^Department of Clinical Oncology, Bristol Cancer Institute, Bristol, UK; ^2^Department of Surgery and Cancer, Imperial College London, London, UK

**Keywords:** tumor metabolism, positron emission tomography, choline, acetate, methionine, glutamine

## Abstract

Cancer cells do reprogram their energy metabolism to enable several functions, such as generation of biomass including membrane biosynthesis, and overcoming bioenergetic and redox stress. In this article, we review both established and evolving radioprobes developed in association with positron emission tomography (PET) to detect tumor cell metabolism and effect of treatment. Measurement of enhanced tumor cell glycolysis using 2-deoxy-2-[^18^F]fluoro-D-glucose is well established in the clinic. Analogs of choline, including [^11^C]choline and various fluorinated derivatives are being tested in several cancer types clinically with PET. In addition to these, there is an evolving array of metabolic tracers for measuring intracellular transport of glutamine and other amino acids or for measuring glycogenesis, as well as probes used as surrogates for fatty acid synthesis or precursors for fatty acid oxidation. In addition to providing us with opportunities for examining the complex regulation of reprogramed energy metabolism in living subjects, the PET methods open up opportunities for monitoring pharmacological activity of new therapies that directly or indirectly inhibit tumor cell metabolism.

## Introduction

Mammalian cells possess molecular machineries that regulate their proliferation, differentiation, and death. Malignant transformation is a multistep process involving genetic alterations, disruption of regulatory circuits, and dynamic changes in the genome. It has been suggested that malignant growth is governed by six essential alterations in cell physiology: self-sufficiency in growth signals, insensitivity to growth-inhibitory (antigrowth) signals, evasion of programed cell death (apoptosis), limitless replicative potential, sustained angiogenesis, and tissue invasion and metastasis (1). Recent advances led to the notion that progressive evolution of normal cells to a neoplastic state involves not only the successive acquisition of hallmark capabilities but also contributions of recruited normal cells (which form tumor-associated stroma, constituting the “tumor microenvironment”) (2).

Metabolic reprograming is an important property of the cancer cells. In the presence of abundant nutrients, oncogenic signaling facilitates assimilation of carbons into macromolecules, such as lipids, proteins, and nucleic acids. The net result of this is to support cell growth and proliferation. Glucose and glutamine are abundant nutrients, and both feed into multiple nodes of central metabolism. Glutamine also provides two nitrogen atoms for synthesis of hexosamines, nucleotides, and amino acids, all of which are also required for growth (3). Among the host of pathways altered in cancer, glucose and glutamine metabolism are consistently reprogramed by mutations in *MYC*, *TP53*, the Ras-related oncogenes, and the LKB1-AMP kinase (AMPK) and PI3 kinase (PI3K) signaling pathways. Oncogenic Ras increases both glucose uptake via enhanced expression of glucose transporter (GLUT) 1, and utilization (4), in addition to regulating glutamine metabolism, promoting cell survival and growth (5). Increased MYC also enhances glycolysis, and glutamine catabolism, resulting in cell growth (6).

The hallmarks of cancer are all linked to proliferation of cancer cells, thus making cell proliferation an important capability leading to immortalization and generation of macroscopic tumors. The framework of hallmarks assumes a homogeneous population of cancer cells and considers the hallmarks as distinct entities, with a one-to-one relation between oncogenic events (the inducers), the signaling pathways (transmission), and the hallmarks (the effects). However, one oncogenic event, or one signaling cascade, could induce several hallmarks accounting for the dynamic and spatial heterogeneity of tumors (7). This heterogeneity provides a framework to interpret pathological, diagnostic, and therapeutic observations of tumors and supports the need for non-invasive serial studies on the whole tumor mass and the use of simultaneous, multi-targeted therapies for treating cancer.

Routine clinical evaluation of cancer therapeutics involves assessment of the change in tumor burden (anatomical measurements). Tumor shrinkage (objective response) and time to disease progression are both important endpoints, as these have been linked to an improvement in overall survival or other time to event measures in randomized phase III studies (8). These measures also determine the efficacy of drugs under study. In order to have standardized and widely accepted criteria for measurement of response to allow comparisons to be made across studies, the Response Evaluation Criteria in Solid Tumors (RECIST) criteria were formulated (9). These criteria have been widely adopted for trials where the primary endpoints are objective response or disease progression. Since the introduction of RECIST in 2000, the increasing utilization of imaging technologies, such as MRI, FDG positron emission tomography (PET), and targeted cytostatic therapies, have prompted an update in the guidelines (RECIST v1.1) (10). The definitions of the criteria used to determine objective tumor response for target lesions are as follows:

1) Complete response (CR): disappearance of all target lesions. Any pathological lymph nodes (whether target or non-target) must have reduction in short axis to <10 mm.
2) Partial response (PR): at least a 30% decrease in the sum of diameters of target lesions, taking as reference the baseline sum diameters.
3) Progressive disease (PD): at least a 20% increase in the sum of diameters of target lesions, taking as reference the smallest sum on study (this includes the baseline sum if that is the smallest on study). In addition to the relative increase of 20%, the sum must also demonstrate an absolute increase of at least 5 mm. (Note: the appearance of one or more new lesions is also considered progression.)
4) Stable disease (SD): neither sufficient shrinkage to qualify for PR nor sufficient increase to qualify for PD, taking as reference the smallest sum diameters while on study.

RECIST has limitations due to its reliance on changes in tumor size with therapy. First, uni-dimensional measurements may be apparent only after three to four cycles of chemotherapy. In non-responders, this means subjecting patients to cumulative toxicity of three to four cycles of treatment with little benefit. Moreover, the change in the tumor diameter may be non-uniform. Second, changes in measurements of smaller lesions are not reliable (11). Third, cytostatic treatments may not necessarily cause a decrease in tumor size or volume. Use of functional imaging overcomes several of these limitations. The use of PET has, for example, resulted in accurate imaging of subtle tumor biologic changes and the detection of early response to anti-cancer therapy (12). Tumors having increased metabolic activity may take up greater amounts of a radioactive tracer as compared to adjacent normal tissues; in that regard, sub-millimeter tumors have been known to show significant radiotracer uptake for certain tracers (13). Similarly, any change in metabolic or signaling pathway activity consequent to successful treatment could result in changes in uptake of the tracer on PET (14). Thus, PET is a useful tool in oncology to image certain metabolic pathways and response to therapy.

**Figure 1:**
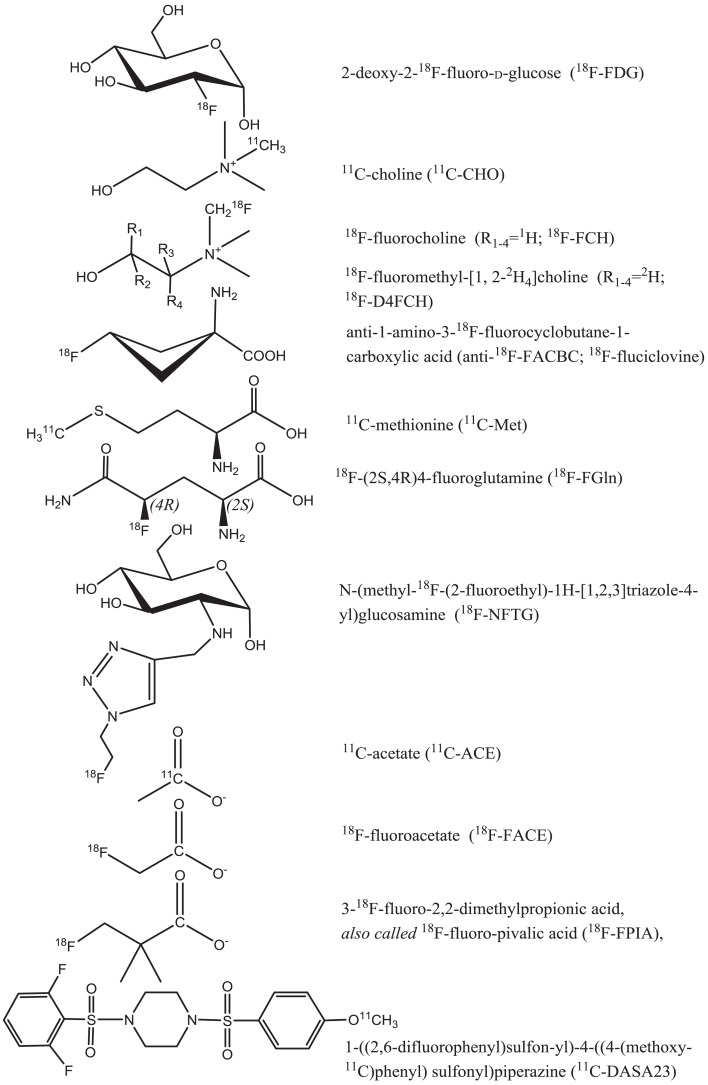
**Radioprobes utilized in the imaging of tumor cell metabolism**.

This review gives an overview of metabolic processes imaged by PET focusing on both established and evolving radioprobes to detect tumor glycolysis, choline metabolism, intracellular transport of glutamine, and other amino acids, as well as fatty acid metabolism (Figure 1). In particular, we emphasize the role of radiolabeled choline, acetate, and amino acid tracers for monitoring efficacy or predicting response to new therapies that directly or indirectly inhibit tumor cell metabolism.

## Methodology

A comprehensive PubMed literature search was performed, identifying articles relating to PET imaging in malignant disease, particularly those reporting on response assessment with radiolabeled tracers, focusing on [^11^C]- and [^18^F]-labeled choline, acetate, methionine, and glutamine derivatives, up to July 2015. Search terms that were used to identify such articles were “acetate,” “choline,” “methionine,” “glutamine,” “tryptophan,” “FACBC,” and “PET” or “positron emission tomography.” Original publications were selected for inclusion in this review.

## Overview of Pathways Traced by Pet Imaging

### Glycolysis and Glycolysis-Linked Metabolic Pathways

A review of metabolism will be incomplete without reference to glycolysis. Energy production in normal cells is predominantly the result of oxidative phosphorylation, as opposed to glycolysis. However, tumor cells predominantly use glycolysis as a means to energy production irrespective of oxygen levels. Aerobic glycolysis (AG) refers to glucose utilization in excess of that needed for oxidative phosphorylation, despite sufficient oxygen to metabolize glucose to carbon dioxide and water. AG plays an important role in the biosynthesis of glycogen, proteins, lipids, and nucleic acids (15). AG, also known as the Warburg effect, supports the biosynthetic requirements of proliferating cancer cells (16). PET using 2-deoxy-2-[^18^F]fluoro-D-glucose ([^18^F]FDG) has been widely used in the evaluation of various tumor types on the basis that an increase in AG will be reflected in an increase in the total glucose consumption of the tissue.

In a large pooled review of over 18,000 patient studies, it was shown that [^18^F]FDG PET has a sensitivity of 84% and a specificity of 88% for tumor detection (17). [^18^F]FDG PET has also been evaluated in response assessment following treatment with conventional chemotherapeutic agents both in the preclinical (18) and in the clinical setting (19–25), with the premise that decreases in glycolysis may occur in tumors that are sensitive to the applied cancer therapeutics and that the tumors that are resistant to treatment will show unchanged glucose metabolism. The prediction of treatment response has also been analyzed in many studies following treatment with different targeted therapies, e.g., monoclonal antibodies and small molecules inhibitors (26). However, [^18^F]FDG PET has the following limitations: (1) False positive uptake in some benign processes, such as infection and inflammatory lesions (27); (2) low sensitivity in well-differentiated/low-grade tumors that have relatively low glucose metabolism such as carcinoid tumors, bronchoalveolar cell carcinoma, and renal cell carcinoma (RCC) (28–30); (3) low sensitivity in hypocellular cancers, such as desmoplastic or mucinous tumors (31); (4) increased [^18^F]FDG accumulation in some normal body regions, such as lymphoid tissue and brown fat (32); and (5) lack of clinical utility due to high urinary excretion and low expression of GLUT in prostate cancer (PCa) (30). Thus, newer radiotracers to image tumors accurately are being developed to address these shortcomings, as well as explore other metabolic pathways of tumors that can be imaged using PET. Two evolving imaging strategies somewhat linked to glycolysis will be discussed next.

Further to the Warburg effect, the final rate-limiting step in AG, catalyzed by pyruvate kinase (PK), controls the balance between energy production and metabolic precursor synthesis. The M2 isoform of PK (PKM2) is preferentially expressed in cancer cells and channels glycolytic intermediates into pentose phosphate pathway for nucleotide synthesis (33). PKM2 can be allosterically regulated to assume a high- or low-activity state. In cancer cells, there is downregulation of PK activity favoring a microenvironment that is conducive to cell proliferation. 1-((2,6-difluorophenyl)sulfonyl)-4-((methoxy-^11^C)phenoxy)sulfonyl)piperazine ([^11^C]DASA-23) (Figure 1) has been developed as a non-invasive PET probe to measure activity of PKM2 in preclinical glioblastoma models (34). Witney and co-workers have demonstrated that [^11^C]DASA-23 improved tumor visualization and predicted response to PKM2 activator, which resulted in loss of PET signal. The clinical translation of these findings is eagerly awaited.

Another glycolysis-linked pathway that has come to the fore is glycogenesis. Glycogen, the principal glucose store in mammalian cells, is synthesized from uridine diphosphate glucose (UDP-glucose) catalyzed by glycogen synthase (GS). Tumor cells originating from epithelial tissues, especially in the quiescent state also accumulate glycogen, in addition to increased glycolytic flux (35, 36). In order to gain biological insight into the role of glycogenesis, PET with [^18^F]-N-(methyl-(2-fluoroethyl)-1H[1,2,3]triazole-4-yl) glucosamine ([^18^F]NFTG) has been studied (37). The authors showed that glycogen levels, [^18^F]NFTG, but not [^18^F]FDG uptake, increased proportionately with cell density and G1/G0 arrest. This increase in glycogen levels and [^18^F]NFTG uptake has potential application in the assessment of oncogenic pathways related to glycogenesis and the detection of post-treatment tumor quiescence. However, there have been no studies evaluating response to therapy.

### Choline Metabolism: Choline PET

Choline is a precursor of phosphatidylcholine (PC), an essential component of phospholipids in the cell membrane (38) and is required for structural stability and cell growth. It is also essential for the synthesis of neurotransmitters such as acetylcholine (by reaction of choline with acetyl-CoA), and production of potent lipid mediators, such as platelet-activating factor. Choline kinase (CHK) is the first enzyme in the Kennedy pathway (39), and is responsible for the *de novo* synthesis of PC. CHK phosphorylates choline to phosphocholine (PCho), the rate-limiting step in the Kennedy pathway. PCho is further phosphorylated to cytidine diphosphate-choline (CDP-choline) by the enzyme cytidylyltransferase and then to other intermediates before being incorporated into cell membrane phospholipids as PC. Malignant transformation is associated with enhanced choline transport and utilization, characterized in a large part by increased CHKα expression, which leads to a phenotype of increased radiolabeled choline uptake (40, 41).

#### Choline Tracers

Choline has been radiolabeled with [^11^C], [^18^F] for tracing choline transport and phosphorylation in several tumor types. In one of the first studies, Hara and colleagues showed that phosphorylation led to intracellular retention of the carbon label [^11^C] in PCa (42), thus enabling imaging of this metabolic pathway. The same group also showed that [^11^C]choline had good uptake in brain tumors with almost negligible activity in the blood after 5 min (43). This work inspired others to use [^11^C]choline as a PET radiotracer to image other tumors, including renal (30), esophageal (44–48), and non-small cell lung cancer (NSCLC) (44). [^11^C]choline is particularly useful in PCa as there is negligible urinary bladder excretion, a challenge with [^18^F]FDG. The utility of [^11^C]choline in visualizing and staging PCa has been extensively reported (42, 49).

[^18^F]Fluorocholine ([^18^F]FCH) was developed to overcome the short physical half-life of carbon-11 (20.4 min). The longer half-life (109.8 min) of [^18^F] was deemed potentially advantageous in permitting late imaging of tumors when sufficient clearance of parent tracer in systemic circulation had occurred. Since DeGrado and co-workers first reported the use of [^18^F]FCH (50), the tracer has proven safe for human administration (51) and has been extensively used in patients for diverse pathologies.

[^11^C]Choline (and fluoro-analog) is, however, readily oxidized to [^11^C]betaine by choline oxidase mainly in kidney and liver tissues, with metabolites detectable in plasma soon after injection of the radiotracer (52–54). This makes discrimination of the relative contributions of parent radiotracer from catabolites difficult when a late imaging protocol is used. A more metabolically stable [^18^F]choline analog, [^18^F]fluoromethyl-[1,2-^2^H_4_]choline ([^18^F]D4-FCH), based on the deuterium isotope effect (55) has been developed. The simple substitution of deuterium [^2^D] for hydrogen [^1^H] and the presence of [^18^F] improve the stability of the compound and reduce degradation of the parent tracer (54, 56, 57). This modification is hypothesized to increase the net availability of the parent tracer for phosphorylation and trapping within cells leading to a better signal-to-background contrast, thus improving tumor detection sensitivity of PET. [^18^F]D4-FCH has been validated for imaging tumors preclinically (56, 57). [^18^F]D4-FCH injection was also found to be safe and well tolerated in healthy volunteers with a favorable dosimetry profile (58). Further clinical studies are now underway to evaluate the utility of [^18^F]D4-FCH in cancer patients.

As the large proportion of studies evaluating response with choline radiotracers has been conducted in PCa – a disease that is managed by a plethora of agents, including androgen deprivation therapy (ADT), radiotherapy (RT), and chemotherapy – this will be the main aspect of the review although other malignancies will be briefly discussed.

#### Preclinical Studies

Radiolabeled choline uptake has been extensively investigated in cells and animal models of cancer to determine factors that affect intrinsic uptake and allow interpretation of clinical findings (Table 1).

**Table 1:** **Response assessment: preclinical studies**.

	Cell lines/animal models	Outcome
**CHOLINE PET**		
**Prostate**		
Hara et al. (59)	LNCaP cells, PC3 cells	Androgen depletion markedly suppressed the uptake of [^3^H]choline in androgen-dependent LNCaP cells but not in androgen-independent PC3 cells
Al-Saeedi et al. (60)	PC3 cells	Flutamide inhibited tumor cell growth and proliferation Flutamide might inhibit proliferation by an androgen-independent mechanism
Holzapfel et al. (64)	LNCaP cells, PC3 cells Dose of RT – 6 Gy	Transient increase in [^3^H]choline uptake seen in PC3 cells (maximum at 24 h). Significant decrease in uptake seen in LNCaP cells (minimum at 48 h)
Krause et al. (62)	PC3 cells, subcutaneous 13 NMRI (nu/nu) mice	Reduction in the mean [^11^C]choline uptake (tumor-to-muscle ratio: TMR) as early as 1 week after initiation of docetaxel [^11^C]choline PET/CT might be a useful tool for monitoring responses to taxane-based chemotherapy
Fei et al. (65)	PC3, CWR22 cells athymic nude mice	For treated tumors, normalized [^11^C]choline uptake decreased significantly 24 and 48 h after photodynamic therapy (PDT), associated with decrease in PSA levels. [^11^C]Choline PET has the potential to determine whether a PDT-treated tumor responds to treatment within 48 h after therapy
Emonds et al. (61)	LNCaP, PC346C cells PC3, PC346DCC cells	Androgens modulated the uptake of [^11^C]choline in PC346C cells but not in PC3 cells Anti-androgen (Bicalutamide) reduced the uptake in PC346C cells
Schwarzenbock et al. (63)	LNCaP cells SCID-mice	[^11^C]choline has the potential for use in the early monitoring of the therapeutic effect of docetaxel
**Breast**		
Al-Saeedi et al. (83)		Incorporation of radiolabeled choline in tumor cells has been shown to be associated with proliferation
**ACETATE PET**		
**Prostate**		
Oyama et al. (94)	CWR22 androgen-dependent cells Nude mice	[^18^F]FDG PET detected metabolic changes within days of androgen ablation in a murine model of prostate cancer, whilst there was no significant difference in [^11^C]acetate uptake
Vavere et al. (96)	LNCaP, PC3, 22Rv1 Male nu/nu mice	Demonstrating that the FASN inhibitor C75 could reduce [^11^C]acetate SUV by up to 60% in prostate cancer xenografts
Yoshii et al. (95)	LNCaP, PC3, 22Rv1, and DU145 cells	Evaluated method to predict FASN-targeted therapy outcome using radiolabeled acetate uptake. They demonstrated that uptake of radiolabeled acetate reflects FASN expression and sensitivity to FASN-targeted therapy with orlistat, indicating uptake of radiolabeled acetate is a useful predictor of FASN-targeted therapy outcome
Emonds et al. (93)	LAPC-4 (androgen sensitive), 22Rv1 cells (androgen-independent) Nude mice	They found that ADT significantly decreased the uptake of [^11^C]choline and [^18^F]FDG but not uptake of [^11^C]acetate after 5d of ADT Concluded that [^11^C]acetate uptake occurs independently of androgens and thus may be more favorable for detecting tumor viability during or following ADT
**METHIONINE PET**		
**Brain**		
Sato et al. (125)	Glioma model	The metabolic changes following intraperitoneal chemotherapy were seen immediately as a sharp fall in [^14^C]thymidine (dThd) and [^18^F]fluoro-2′-deoxyuridine ([^18^F]FUdR) uptake and a moderate fall for [^14^C]methionine whereas decrease in [^3^H]deoxyglucose (DG) were seen 1 week after chemotherapy
Reinhardt et al. (123)	AH109A hepatoma cells Donryu rats	[^11^C]Methionine PET has been sensitive enough to detect and differentiate viable cancer cells in a residual tumor mass as compared to FDG and thymidine, 6 days after one to eight doses of 5 Gy ^60^Co radiotherapy (RT)
Sasajima et al. (124)	Glioma C6 and C6R cells *In vitro and in vivo* Sprague-Dawley rats	The [^3^H]TdR accumulation rate and amino acid tracer trans-1-amino-3-fluoro-1[^14^C]-cyclobutanecarboxylic acid [^14^C]FACBC and [^3^H]Met uptake significantly decreased 48 and 72 h, respectively, after temozolomide (TMZ) treatment in C6 but not C6R cells. The decrease in uptake was seen before morphological changes on MRI. Anti-[^14^C]FACBC and [^3^H]Met could be a sensitive and precise imaging biomarker for tumor extent visualization and response assessment in glioma patients.
Ono et al. (121)	Human Glioblastoma, U87MG (U87) cells U87 and U87R F344/N-mu rats	PET with amino acid tracers (1-amino-3-[^18^F]fluorocyclobutanecarboxylic acid ([^18^F]FACBC) and [^11^C]Methionine) provides useful information on the early response of glioblastomas to single-agent [TMZ, interferon-β (IFN), and bevacizumab (Bev)] and combination therapy in glioblastoma
**Breast**		
Paquette et al. (122)	MC7-L1 (ER+) and MC7-L1 ERα-knockdown cell lines Balb/c mice	Letrozole and Fulvestrant reduced glucose uptake/consumption (FDG) and protein synthesis ([^11^C]Methionine) in ER+ tumors, but not so in ERαKD tumors
**Radiotherapy effect**		
Kubota et al. (118)	AH109A hepatoma cells Donryu rats	A rapid reduction in [^11^C]methionine uptake following therapy in animal studies was demonstrated
Schaider et al. (126)	SW707 colon cancer cells	In an experimental tumor model, MET uptake showed a rapid decrease after irradiation and was followed by necrosis and progressive tumor shrinkage
Murayama et al. (120)	SCCV11, murine squamous cell carcinoma cell line C3H/HeN mice	Tumor uptake was decreased with all the tracers (FDG, [^11^C]Methionine, FLT, [^18^F]FMT) after were treated with a single dose of x-ray irradiation at 2, 6, 20, or 60 Gy. Significant positive correlations were found between ligand uptake and tumor volume for [^18^F]FMT
**Gynecological**		
Higashi et al. (116)	Human ovarian carcinoma cell line (HTB77IP3)	Early assessment of human adenocarcinoma response to radiotherapy by FDG, Thymidine, and [^11^C]methionine PET may be confounded by a normal increase in tracer uptake post-irradiation (30 Gy ^60^Co irradiation), despite a 6.25-fold decline in viable cell numbers
Trencsenyi et al. (127)	A2780AD/A2780 human ovarian carcinoma and KB-V1/KB-3-1 human epidermoid adenocarcinoma tumor CB-17 SCID mice	FDG, FLT, [^11^C]Methionine and [^18^F]fluoroazomycin-arabinofuranoside ([^18^F]FAZA) are suitable PET tracers for the diagnosis and *in vivo* follow-up of the efficacy of tumor chemotherapy (doxorubicin) in both Pgp(+) and Pgp(−) human tumor xenografts by mini PET
**Myeloma**		
Luckerath et al. (119)	OPM2, MM.1S myeloma cell lines NOD.CB17-*Prkdc^scid^*/NCrHsd mice	[^11^C]Methionine is superior to FDG (30–79% reduction in [^11^C]Methionine uptake) in very early assessment (24h post) of response to Bortezomib

Hara and colleagues demonstrated that androgen depletion markedly suppressed the uptake of [^3^H]choline in androgen-dependent LNCaP cells but not in androgen-independent PC3 cells (59). Anti-androgens were subsequently shown to modulate choline uptake in androgen-dependent cell lines and also inhibit proliferation (60, 61). Regarding chemotherapy, the effects of docetaxel have been studied by Krause et al. (62), who showed a reduction in the mean [^11^C]choline uptake in PC3 xenograft mouse model as early as 1 week after initiation of docetaxel. A significant reduction of mean tracer uptake of 45% was associated with a mean tumor growth inhibition of 20%. They concluded that [^11^C]choline might be a useful tool for monitoring responses to taxane-based chemotherapy in patients with advanced PCa. These findings were confirmed by Schwarzenbock et al. in a LNCaP-xenograft mouse model (63). Thus, labeled choline uptake is intrinsically responsive to anti-androgen therapy and chemotherapy.

Regarding RT, Holzapfel et al. have studied the effect of 6 Gy of radiation on PC3 and LNCaP cells, *in vitro* (64). Radiation-induced effects were variable with a transient increase in radiotracer uptake in androgen-independent PC3 cells but a decrease in androgen-dependent LNCaP cells. In both cell lines, modulation of tracer uptake was dose-independent following irradiation with 2–12 Gy with a mean increase to 120% in PC3 cells and a mean decrease to 74% in LNCaP cells. The authors suggested that changes of tumor choline uptake monitored by PET after RT may be due to a combination of factors, including therapeutic efficacy and altered tracer transport in cancer cells as a consequence of irradiation. Photodynamic therapy (PDT) responses have also been evaluated. Fei and co-workers evaluated the potential use of [^11^C]choline PET to monitor early tumor response to PDT in animal models. For treated tumors, normalized [^11^C]choline uptake decreased significantly at 24 and 48 h after PDT, associated with decreases in prostate-specific antigen (PSA) levels. The authors concluded that [^11^C]choline PET has the potential to determine response in a PDT-treated tumor within 48 h after therapy (65).

#### Clinical Studies

To date, only anecdotal reports (50, 66) and two small clinical studies (67, 68) have assessed the role of [^11^C]choline PET as a method to monitor the therapeutic effects of ADT (Table 2). Fuccio et al. (67) retrospectively evaluated the effect of 6 months of androgen deprivation (Zoladex in 12 and Bicalutamide in 2 patients) in 14 PCa patients with recurrence after radical prostatectomy. They concluded that androgen deprivation significantly decreases [^11^C]choline uptake in androgen-sensitive patients. In another study in six primary PCa patients having bicalutamide therapy, Giovacchini et al. (68) showed an average reduction of 45% in the [^11^C]choline uptake (SUV_max_ from 11.8 to 6.4) corresponding to a 78% decrease in PSA following a median of 4 months of therapy. A similar magnitude of reduction in SUV_ave_ and SUV_max_ in the prostate tumors corresponding to 94% reduction in PSA was shown by Challapalli and co-workers, in patients having neoadjuvant ADT (69).

**Table 2:** **Choline PET response assessment: clinical studies**.

	Sample size	Outcome
**CHOLINE PET/CT**		
**Prostate**		
De Grado et al. (50)	1	60% reduction in choline uptake in the primary tumor and the bony metastases with androgen deprivation therapy (ADT) in patient with bone metastases from PCa
Giovacchini et al. (68)	6	45% reduction in the [^11^C]choline uptake (SUV_max_) from 11.8 to 6.4 with a 78% decrease in PSA with a median of 4 months of bicalutamide therapy in patients with primary prostate cancer
Beheshti et al. (72, 73)	38	Demonstrated that reduced [^18^F]FCH uptake is seen in PCa patients who respond to the hormone therapy often without any significant morphological CT changes
De Waele et al. (66)	1	Initial uptake in prostate and multiple iliac nodes in locally advanced disease, disappeared after 6 months of therapy with leuprorelin and flutamide
Fuccio et al. (67)	14	Six months of androgen deprivation significantly decreases [^11^C]choline uptake in patients with recurrence after radical prostatectomy
Casamassima et al. (70)	25	High dose of radiotherapy is effective in eradication of limited nodal recurrences
Kwee et al. (76)	8	Plasma cfDNA content and FCH PET/CT-detected tumor activity are potential candidate markers of therapeutic response in castrate resistant prostate cancer (CRPC)
Amani et al. (71)	11	Intra-prostatic [^11^C]choline uptake (as measured by SUV_max_ and TMR) significantly decreased during and after RT
Challapalli et al. (69)	10	[^11^C]choline uptake in prostate tumors, determined by [^11^C]choline PET/CT, is sensitive to ADT and RT, and could be used as an objective quantitative tool for response assessment
De Giorgi et al. (79)	43	Early FCH PET/CT can predict clinical outcome (Progression free and overall survival: PFS and OS) than PSA response in patients on Abiraterone
Caffo et al. (77)	31	Enzalutamide induces volume reductions in primary tumors and metabolic changes in metastatic lesions as detected by [^18^F]FCH PET/CT
De Giorgi et al. (78)	36	Combination of changes in [^18^F]FCH PET/CT and decrease in PSA level in patients on enzalutamide could be a valid tool to predict PFS in metastatic CRPC patients
Miyazaki et al. (80)	2	[^18^F]FCH PET/CT detected changes in bone metastatic activity midway during treatment with radium-223 dichloride. Whole-body tumor burden decreased in one patient, while a heterogeneous tumor response was observed in the other. Corresponding normalization and persistent elevation in serum alkaline phosphatase levels were observed in these cases, respectively
**Renal cell cancer**		
Middendorp et al. (86)	2	[^18^F]FEC PET/CT before and 10 weeks after two cycles of tyrosine kinase inhibitor therapy showed progression in one patient and partial response in the other
**Brain**		
Parashar et al. (81)	14 (various tumor sites)	[^18^F]FCH PET/CT is potentially a predictive biomarker for early detection (after 3–4 weeks) of RT/CRT response in patients with lesions in base of tongue, tonsil, nodes, hypopharynx, maxilla, palate, lung, pancreas, brain, uterus, and rectum with 88% patients had response (complete and partial response: CR and PR)
Panagiotidis et al. (82)	1	Simultaneous PET/MRI with [^18^F]choline in a patient with pineal germ cell tumor demonstrated a reduction in both size and radiotracer activity of the mass after chemotherapy
**Breast**		
Kenny et al. (85)	2	[^11^C]choline uptake was lower in two patients responding to trastuzumab treatment, suggesting that [^11^C]choline PET may be useful in detecting the response of breast cancer to trastuzumab treatment

**Figure 2:**
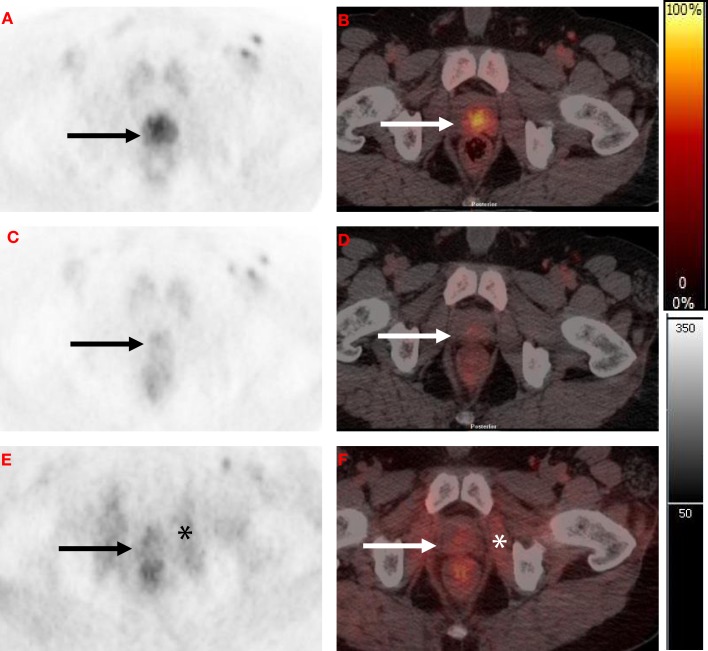
**Axial [^11^C]choline PET and fused PET/CT at level of the prostate**. **(A,B)** Baseline scan with focal activity in the peripheral zone (black and white arrows). **(C,D)** Post-neoadjuvant androgen deprivation therapy (NAD) scan (8–10 weeks after initiating NAD) with a marked reduction in [^11^C]choline uptake in the peripheral zone. **(E,F)** Post- radiotherapy combined with concurrent androgen deprivation therapy (RT-CAD) scan (4 months after completion of RT-CAD) with a further reduction in prostate activity and increased obturator internus muscular activity (black and white asterisk).

There is paucity of data on use of [^11^C]choline PET to monitor response to RT. Based on 2-month post-RT [^11^C]choline PET/CT reductions, Casamassima and colleagues inferred that high-dose RT was effective in eradication of limited nodal recurrences (70). More recently, in a study of 11 patients with intermediate-risk PCa, Amani and co-workers evaluated sequential [^11^C]choline PET/CT scans before and up to 12 months after completion of RT (74 Gy/37 fractions). None of the patients received hormonal therapy. They concluded that RT significantly decreased intra-prostatic [^11^C]choline uptake (as measured by SUV_max_ and tumor-to-muscle ratio (TMR) (71). Thus, the concern that RT might increase labeled choline transport does not appear to occur in patients at clinically relevant doses. Furthermore, in a proof of concept study, Challapalli and co-workers showed that choline uptake in prostate tumors, determined by [^11^C]choline PET/CT, is sensitive to ADT and RT, and could be used as an objective quantitative tool for response assessment. ADT decreased tumor-imaging variables – SUV_60,ave_, SUV_60,max_, TMR_ave_, and TMR_max_ – by 26–60%. RT also decreased [^11^C]choline uptake within primary prostate tumors (though of lesser magnitude: 12–27%), compared to that seen with ADT, except for TMR_max_ where a significant reduction (40%) was seen (Figure 2) (69). Similarly, Beheshti and colleagues demonstrated that reduced [^18^F]FCH uptake is seen in PCa patients who respond to the hormone therapy often without any significant morphological CT changes (72, 73). These studies show the potential of radiolabeled choline to detect response of early PCa to therapies used routinely in the clinic.

Chemotherapy, Radium-223, and drugs interfering with androgen receptor (AR) machinery, such as enzalutamide and abiraterone, are the main stay of treatments in metastatic castrate resistant prostate cancer (mCRPC). Currently, there is excessive reliance on changes in serum PSA as an indicator of therapeutic efficacy and there are no predictive diagnostic tools to identify an early objective response in patients with mCRPC treated with abiraterone acetate or enzalutamide, although AR splice variants detectable in circulating tumor cells (CTCs) are evolving (74). The Prostate Cancer Clinical Trials Working Group recommends waiting 12 weeks before the first evaluation of response to ensure adequate drug exposure (75). Therefore, studies investigating new biomarkers for outcome prediction and disease monitoring are urgently needed. To this effect, labeled choline PET is under evaluation to assess therapeutic response.

Kwee and colleagues evaluated effects of docetaxel chemotherapy on circulating cell-free DNA (cfDNA) and [^18^F]FCH PET/CT uptake in CRPC. Tumor-derived plasma cfDNA concentrations increased significantly after one and three treatment cycles, possibly due to post-chemotherapy necrotic cell lysis. Lower cfDNA concentrations at baseline were found to be associated with PET responses as measured after the third chemotherapy cycle. They concluded that it is feasible to annotate potential tumor sources of cfDNA using [^18^F]FCH PET/CT imaging, and that plasma cfDNA content and [^18^F]FCH PET/CT-detected tumor activity are potential response markers in CRPC (76). Caffo et al. showed that enzalutamide induces volume reductions in primary tumors and metabolic changes in metastatic lesions as detected by [^18^F]FCH PET/CT (77). In a proof of principle study, De Giorgi et al. evaluated [^18^F]FCH PET/CT as an early predictor of outcome in mCRPC patients treated with enzalutamide (78). They concluded that the combination of [^18^F]FCH PET/CT and decrease in PSA level could be a valid tool to predict progression-free survival (PFS) in mCRPC patients.

In a similar study with abiraterone, De Giorgi et al. demonstrated that early [^18^F]FCH PET/CT can predict clinical outcome (PFS and overall survival: OS) than PSA response in patients on abiraterone. Using fairly arbitrary thresholds for change in SUV (as specified in European Organization for Research and Treatment of Cancer (EORTC) guidelines), a PSA decline ≥50% was shown to be associated with the [^18^F]FCH PET/CT response (12/42)/non-response (18/42) in 71% of patients (79). Miyazaki and co-workers evaluated acute changes in net metabolically active tumor volume (MATV) and total lesion activity (TLA) detected by [^18^F]FCH PET/CT imaging midway during treatment with radium-223 dichloride, in two patients. After the third dose of radium-223 dichloride, near-total disappearance of abnormal skeletal activity was observed in one case, while a heterogeneous tumor response was observed in the other (80). It can be summarized that, while being a proliferation-independent phenotype (13), changes in labeled choline uptake reflects response to therapy although the optimal time still needs to be clarified.

#### Non-Prostate Tumors

In addition to PCa, radiolabeled choline has been utilized in other tumor histotypes. Parashar et al. explored whether [^18^F]FCH PET could serve as an predictive biomarker for early detection of RT/chemo-radiotherapy (CRT) response in patients with lesions at the base of the tongue, tonsil, nodes, hypopharynx, maxilla, palate, lung, pancreas, brain, uterus, and rectum. They demonstrated reductions in SUV_max_ in 88% of lesions (CR: 7/16 and PR: 7/16) and concluded that changes in SUV_max_ after 3–4 weeks of initiation of RT were predictive of final outcome (81). Panagiotidis and co-workers showed that simultaneous PET/MRI with [^18^F]FCH in a patient with pineal germ cell tumor demonstrated a reduction in both size and radiotracer activity of the mass after chemotherapy (82).

While the choline phenotype has been reported as being proliferation independent in PCa (13), the phenotype is intrinsically associated with proliferation in breast cancer cells (83). In particular, PCho formation is linked to the activity of mitogen-activated protein kinase (MAPK) signaling function (84). It was, thus, postulated that therapy response in breast cancer might be accompanied by predictable changes in the tumor retention of [^11^C]choline. In a clinical study involving breast cancer patients receiving trastuzumab, [^11^C]choline uptake decreased in two patients responding to trastuzumab compared to non-responders (85). Regarding targeted therapies, Middendorp et al. also evaluated use of [^18^F]fluoroethylcholine (FEC) PET/CT in staging and monitoring therapy response of advanced RCC before and 10 weeks after two cycles of tyrosine kinase inhibitor (TKI) therapy. FEC PET/CT showed heterogeneous changes, with progression in one patient and a PR in the second patient, which were concordant with the RECIST response (86). Thus, changes in uptake of labeled choline in non-prostate histotypes also appear to reflect therapy response.

### Fatty Acid Metabolism

#### Fatty Acid Synthesis: Acetate PET

Cancer cells are dependent on their ability to gain access to energy and substrate precursors, by reprograming the normal metabolic pathways required for the proliferation and survival of tumor cells, to synthesize of proteins, nucleotides, and lipids (87). Cancer cells are also characterized by a lipogenic phenotype (88), and often require that fatty acids be generated *de novo* to maintain proliferation and viability. As a result, fatty acid biosynthesis has gained significance as a potential therapeutic target in multiple cancers.

Acetate is metabolized in the tricarboxylic acid (TCA) cycle yielding CO_2_ and water (89). However, in cancer cells, acetate is preferentially utilized for fatty acid synthesis as a component of acetyl-CoA. Intracellularly, acetate is converted to acetyl-CoA by acetyl-CoA synthase (ACeS), and fed into the fatty acid synthesis pathway. [^11^C]Acetate PET was originally used to assess myocardial function (90). In myocardial tissues, carbons derived from [^11^C]acetate are incorporated into CO_2_ during the TCA cycle, allowing for PET visualization of oxygen consumption. However, in tumor cells, [^11^C]acetate is incorporated into membrane lipids due to over-expression of fatty acid synthase (FASN). This property is exploited for tumor imaging with [^11^C]acetate (91). The majority of studies analyzing the efficacy of [^11^C]acetate PET in tumors have focused on the detection of PCa (91). In addition to PCa, there are a number of other tumor types in which [^11^C]acetate PET shows high contrast, including hepatocellular carcinoma (HCC), thymomas, renal cancers, brain tumors, and bronchioloalveolar carcinoma (92). These studies demonstrate that [^11^C]acetate is useful in diagnosis, staging, and predicting disease progression in certain cancers, and it is logical that [^11^C]acetate could also be used to stratify patients for specific therapies, as well as a method to monitor response to therapy.

##### Preclinical Studies

Emonds et al. evaluated the effect of 5 days of ADT on the uptake of [^11^C]acetate, together with [^18^F]FDG and [^11^C]choline *in vivo*. They found that ADT significantly decreased the uptake of [^11^C]choline and tended to decrease [^18^F]FDG uptake. [^11^C]Acetate uptake was unaffected by ADT in both PCa xenograft models [LAPC-4 (androgen sensitive), 22Rv1 cells (androgen-independent)]. The authors concluded that [^11^C]acetate uptake occurs independently of androgens and, thus, may be more favorable for detecting tumor viability during or following ADT (93). These findings were corroborated by Oyama et al. who also showed that [^18^F]FDG PET, detected metabolic changes within days of androgen ablation, while there was no significant difference in [^11^C]acetate uptake in a murine model of PCa (94).

[^11^C]Acetate PET could potentially be used as a surrogate for monitoring FASN activity as the incorporation of [^11^C]acetate into membrane lipids is regulated by FASN expression. There is potential that this approach may be an effective means to validate FASN inhibitors as they progress through clinical development. Yoshii et al. (95) evaluated a method to predict FASN-targeted therapy outcome using radiolabeled acetate uptake in LNCaP, PC3, 22Rv1, and DU145 cells. They demonstrated that uptake of radiolabeled acetate reflects FASN expression and sensitivity to FASN-targeted therapy with orlistat. Furthermore, Vavere et al. demonstrated that the FASN inhibitor C75 could reduce [^11^C]acetate uptake by up to 60% in PCa xenografts (96). While these studies are promising (Table 1), it has been noted recently that optimal acquisition of [^11^C]acetate images may require late imaging (~90 min) to increase sensitivity toward lipid incorporation (97).

##### Clinical Studies

Yu and co-workers, tested the feasibility of [^11^C]acetate PET imaging to assess response to therapy in men with bone metastatic PCa. Patients were imaged before and 6–12 weeks after initial ADT for new metastatic PCa or after first-line chemotherapy with docetaxel for CRPC. Changes in qualitative assessment and tumor:normal uptake ratio correlated with clinical response criteria. They concluded that [^11^C]acetate PET scanning was highly accurate for determining response to treatment in patients with bone metastases (98). Regarding therapy planning, Gomez and co-workers reported that [^11^C]acetate PET aids in detection of lymph node metastases especially in high risk PCa patients and led to changes of radiation therapy treatment field/volume or dose in about one-third of the patients (37%). Changes in [^11^C]acetate may serve as a tool for monitoring radiation therapy response (99).

There are anecdotal reports of [^11^C]acetate PET monitoring response in RCC and meningiomas. Maleddu et al. suggested that [^11^C]acetate PET could predict response to sunitinib as early as 2 weeks after therapy initiation (100). In the evaluation of meningiomas, Liu RS et al. demonstrated that [^11^C]acetate had good sensitivity for detection of meningiomas compared to [^18^F]FDG, and concluded that [^11^C]acetate performed better in monitoring five patients who had received gamma-knife surgery (101). [^11^C]acetate PET has also been used in assessing response to therapy in multiple myeloma. Bone marrow histology and whole-body (WB) MRI were used as comparators. In 13 patients who had repeat examination after induction therapy, visual and quantitative analysis, suggested a higher detection rate for both diffuse and focal myeloma lesions at diagnosis. After treatment, a 66% reduction in SUV_max_ was seen in patients with at least a very good PR (≥ 90% reduction in M-protein) versus a 34% reduction in those with a PR (≥50% reduction in M-protein). They concluded that [^11^C]acetate may be valuable for response assessment (102). In aggregate, the initial data with [^11^C]acetate for monitoring response is promising (Table 3) but the short half-life of the tracer may reduce sensitivity for imaging lipid incorporation, and its use is limited to centers with in-house cyclotron. Moreover, acetate is not specific to fatty acid synthesis, it also serves as a substrate for protein acetylation, and is utilized in cholesterol synthesis.

**Table 3:** **Acetate PET response assessment: clinical studies**.

	Sample size	Outcome
**Renal cell cancer**		
Maleddu et al. (100)	1	[^11^C]acetate PET could predict response to sunitinib as early as 2 weeks after therapy initiation
**Brain**		
Liu et al. (101)	22	[^11^C]acetate had a good sensitivity in detection, of meningioma compared to [^18^F]FDG. [^11^C]acetate performed better in monitoring five patients who had received gamma-knife surgery
**Prostate**		
Yu et al. (98)	6	[^11^C]acetate PET scanning was highly accurate for determining the response to treatment in prostate cancer patients with bone metastases
Gomez et al. (99)	19	Changes in [^11^C]acetate may serve as a tool for monitoring radiation therapy response in high risk prostate cancer
**Myeloma**		
Lin et al. (102)	15	Visual and quantitative analysis showed a higher detection rate of myeloma lesions at diagnosis than using [^18^F]FDG. After treatment, a 66% reduction in SUV_max_ was seen in patients with at least a very good partial response versus a 34% reduction in those with a PR. They concluded that [^11^C]acetate may be valuable for response assessment

##### [^18^F]Fluoroacetate

[^18^F]Fluoroacetate ([^18^F]FACE), a [^18^F]fluorinated acetate analog (t1/2: 110 min), which is putatively converted to fatty acids and incorporated into lipids, has been tested as an alternative PET tracer for imaging fatty acid synthesis. However, there are only limited clinical reports using [^18^F]FACE for oncologic diagnosis of patients with cancer (103, 104), thus far with no studies evaluating therapy response.

#### Fatty Acid Oxidation: [^18^F]Fluoropivalic Acid PET

In addition to fatty acid synthesis, the critical nature of fatty acid oxidation for cancer cells survival has been recognized (105). Short-chain carboxylates, including acetate, propionate, butyrate, and the non-natural substrate pivalate (trimethylacetate) use the early steps of the fatty acid oxidation pathway involving acyl-CoA and acyl-carnitine synthesis (106). A new radioprobe, 3-^18^F-fluoro-2,2-dimethylpropionic acid, also called [^18^F]fluoropivalic acid ([^18^F]FPIA), for imaging the early steps of the fatty acid oxidation pathway has been validated and has shown promise for cancer detection (107). Further studies are eagerly awaited.

### Amino Acid Metabolism

Amino acids play an important role in the synthesis of a variety of nitrogen-containing compounds, such as proteins and nucleotides during cell growth, and their increased transport and utilization are be associated with early events in carcinogenesis (108). Natural amino acids are transported into cells by specific carrier-mediated transport systems and further incorporated into proteins and intermediary metabolites to different extents. Thus, investigators have studied the utility of several radiolabeled natural amino acids (including methionine, glycine, tyrosine, phenylalanine, and leucine) as tumor-imaging agents with PET (109). Amino acid scanning provides higher contrast between tumor and normal brain compared to [^18^F]FDG PET, due to the low uptake of amino acids in normal brain. However, of the several amino acid tracers investigated for tumor imaging, only a few have been evaluated beyond the initial feasibility studies in human patients.

#### Glutamine

Glutamine is the most abundant amino acid in plasma and occupies a unique niche in intermediary metabolism by providing a major inter-organ shuttle for both nitrogen and carbon (110). This makes it essential for cell proliferation by contributing to synthesis of nucleic acids, proteins, and hexosamines. Loss of amino and amido groups in glutamine produces alpha-ketoglutarate that also promotes cell growth, anaplerosis and adenosine-tri-phosphate (ATP) generation (111). Malignant transformation, involving enhanced c-Myc expression, increases glutamine metabolism by increased expression of cell surface transporters (112, 113).

Glutamine metabolism lends itself to evaluation by PET imaging, most relevant in non-[^18^F]FDG avid tumors, such as prostate, bronchoalveolar carcinomas, carcinoid tumors, and low-grade lymphomas. These malignancies may use glutamine as an alternative nutrient source and as such are more easily detected by a glutamine-based tracer. Venneti and co-workers, for example demonstrated that PET imaging *in vivo* with the glutamine analog 4-[^18^F]-(2S,4R)-fluoroglutamine ([^18^F]FGln) facilitates clear tumor delineation due to high tumor-to-background ratio. Chemo/radiation therapy reduced [^18^F]FGln tumor uptake, which was associated with decreased tumor burden, confirmed on autoradiography. In contrast, there were no anatomical or structural changes seen on T2-weighted MRI sequences, within the same time frame. These findings were translated into humans (six patients) and an increased [^18^F]FGln uptake was seen in patients with progressive brain tumors, but not in patients with SD (114).

#### Methionine

Methionine, an essential sulfur amino acid, is necessary for growth and development. It plays an important role in protein synthesis and is a predominant methyl group donor for multiple metabolic pathways. Malignant transformation enhances demand for methionine in cancer cells caused by increased fluxes in the pathways of protein synthesis, transmethylation, and transsulfuration. This forms the basis for higher uptake of labeled methionine in tumors.

Currently, PET using L-[methyl-^11^C]-methionine ([^11^C]methionine) is the most popular amino acid imaging modality for tumors, although its use is restricted to PET centers with an on-site cyclotron facility. [^11^C]methionine PET has been extensively studied in gliomas. Its role in initial diagnosis, differentiation of tumor recurrence from radiation injury, grading, prognostication, tumor extent delineation, biopsy planning, surgical resection and RT planning has been evaluated (115). A number of oncologic imaging studies have evaluated the role of [^11^C]methionine in response assessment and have been described in detail in the preclinical setting (116–127) (Table 1) and in patients (128–170) (Table 4). While most studies have focused on non-hematological solid tumors, multiple myeloma also represents an evolving area of interest. In this case, preclinical studies demonstrate superiority of [^11^C]methionine to [^18^F]FDG in monitoring novel anti-myeloma therapy involving proteasome inhibition (119).

**Table 4:** **Methionine PET response assessment: clinical studies**.

	Sample size	Outcome
**Brain**		
Bergstrom et al. (128)	400	In a large series of pituitary adenomas and in some meningiomas, a decrease in the uptake of [^11^C]methionine after medical therapy has been shown to represent a positive treatment effect. [^11^C]methionine PET method does have potential for the evaluation of treatment effects
Kubota et al. (117)	70	[^11^C]Methionine seemed to have a higher potential for rapid tumor monitoring than FDG after radiotherapy, and the effect was radiation-dose dependent
Sato et al. (159)	1	Serial [^11^C]methionine PET imaging in low-grade astrocytoma permits evaluation of changes after radio-chemotherapy treatment in patients in whom CT has revealed no notable changes
Wurker et al. (169)	5	A dose-dependent decline in [^11^C]methionine uptake with a greater decrease in tumors with high basal uptake of [^11^C]methionine
Voges et al. (167)	10	One year after seed implantation of ^125^I for brachytherapy in treatment of cerebral glioma, there were no changes in glucose metabolism, but a significant decline of [^11^C]methionine uptake was seen [^11^C]methionine PET may improve tumor delineation, and allows monitoring of therapeutic effects following brachytherapy
Roelcke et al. (158)	30	No significant difference in [^11^C]methionine and [^18^F]FDG tracer uptake between tumors with or without adjuvant radiotherapy after surgery for low-grade astrocytomas
Shintani et al. (161)	1	Serial [^11^C]methionine PET in a biopsy-proven case of gliomatosis cerebri (GC) suggested initial hypermetabolism, associated with increase in cerebral blood flow (shown on [^15^O]water PET) that normalized 6 months after completion of radiotherapy
Nuutinen et al. (155)	13	[^11^C]methionine PET improves tumor visualization in patients with low-grade glioma and signifies better prognosis in patients with low tumor uptake at baseline. Stable or decreasing uptake of [^11^C]methionine in tumor area after radiotherapy signifies a favorable outcome
Gudjonsson et al. (135)	19	Stereotactic proton beam irradiation of meningiomas had an inhibitory effect (average 19.4% reduction in uptake after 36-month of follow-up) on the [^11^C]methionine uptake in meningiomas, although tumor size remained unchanged (CT/MRI)
Sorensen et al. (162)	2	A prompt reduction in [^11^C]methionine uptake was seen within d of starting therapy in two children with prolactinomas
Muhr et al. (152)	12	During IFN-alpha treatment, [^11^C]methionine PET demonstrated a mean relative percentage of reduction in the uptake ratio (MRelR) of 22.3% in meningiomas
Herholz et al. (137)	1	Estimated a reduction rate in [^11^C]methionine defined active tumor volume of approximately 2.4% per day in a case of anaplastic oligoastrocytoma after procarbazine, CCNU, and vincristine (PCV) chemotherapy
Tang et al. (164)	7	A significant reduction in [^11^C]methionine uptake and a semiquantitative index based on both [^11^C]methionine uptake and [^11^C]methionine defined volume was noted in low-grade oligodendroglioma patients after chemotherapy with PCV regime. Prediction of long-term outcome and effect on high-grade gliomas could not be assessed
Ribom et al. (157)	32	[^11^C]methionine PET may be a promising surrogate endpoint after treatment of grade II gliomas. An increase in [^11^C]methionine uptake or [^11^C]methionine defined volume on follow-up scans was associated with a reduced time to progression of disease in patients with histologically confirmed supratentorial WHO grade II gliomas
Nariai et al. (153)	194	Patients with high-grade glioma showed a significantly decreased post-irradiation tumor-to-normal tissue ratio of [^11^C]methionine uptake compared with the pre-treatment value
Galldiks et al. (131)	15	[^11^C]methionine PET performed before and after the third cycle of temozolomide (TMZ) chemotherapy in patients with malignant gliomas, showed a significantly longer median time to progression in patients with decline in [^11^C]methionine uptake than in those with increasing [^11^C]methionine uptake (23 versus 3.5 months)
Kawai et al. (143)	3	[^11^C]methionine PET findings suggested presence of increased tumor activity in patients with germinomas in the basal ganglia or thalamus after the initial treatment, which gradually decreased during the course of intensive therapy in these patients
Galldiks et al. (132)	1	[^11^C]methionine PET metabolic activity showed a continuous decline of tumor volume, over a 2-year period, below the threshold of significant [^11^C]methionine uptake in patient with glioblastoma multiforme (GBM), treated with surgery, radiosurgery, and maintenance of imatinib and hydroxyurea
Lee et al. (146)	3	A gradual decrease of [^11^C]methionine uptake in basal ganglia germinoma during the course of treatment was seen but the temporal pattern of [^11^C]methionine uptake during the treatment was not evaluated
Jang et al. (140)	4	After high-dose methotrexate chemotherapy for primary CNS Lymphoma (PCNSL), [^11^C]methionine PET displayed complete disappearance of abnormal uptake in all four patients, corroborated on post-treatment MRI and clinical follow-up in three patients
Galldiks et al. (133)	1	A continuous decline in metabolically active tumor volume after stereotaxy-guided laser-induced interstitial thermotherapy (LITT) was observed in a patient with a recurrent GBM, suggesting that [^11^C]methionine PET could be useful for monitoring the short-term therapeutic effects of LITT
Miwa et al. (151)	42	Metastatic lesions demonstrated significant decreases in [^11^C]methionine uptake (quantitative analysis) following stereotactic radiation therapy with intensity modulated radiation therapy (SRT-IMRT: 25–35 Gy in five fractions) in metastatic brain tumors
Chiba et al. (130)	14	A voxel-wise parametric response map (PRM) analysis of [^11^C]methionine PET could be useful for monitoring treatment response in immunotherapy for malignant gliomas
**Head and neck**		
Lindholm et al. (150)	15	In patients with squamous cell carcinomas of the head and neck region treated with preoperative radiotherapy (dose of 61–73 Gy), [^11^C]methionine PET demonstrated a significantly lower [^11^C]methionine uptake in tumors showing a histopathological response when examined before and 5–42 days after radiotherapy
Nuutinen (154)	15	A significant decrease in [^11^C]methionine uptake was seen during the first 2–3 weeks after radiotherapy of head and neck cancer, but the rate of decrease in tracer uptake could not distinguish between relapsing disease and locally controlled disease
Chesnay et al. (129)	13	Reduction in [^11^C]methionine PET accumulation after the completion of one course of chemotherapy for hypopharynx squamous cancer correlated significantly with a reduction in the tumor mass, as measured by MRI at the completion of three courses of chemotherapy
Hasebe et al. (136)	39	[^11^C]methionine PET allowed for a prediction of the therapeutic efficacy of carbon-ion radiotherapy (CIRT) in head and neck adenocarcinomas. Tumor-to-normal tissue ratio pre-treatment (TNRpre) was significantly associated with metastasis and disease-specific survival, while the TNR post-treatment (TNRpost) was associated with the local recurrence, metastasis, and disease-specific survival
Toubaru et al. (165)	67	[^11^C]methionine PET or PET/CT prior to and 1 month after the completion of CIRT for adenoid cystic carcinoma of the head and neck, showed a significant decrease in TNR after treatment
**Breast**		
Huovinen et al. (138)	8	A reduction in [^11^C]methionine uptake predicted clinical target stability or regression of metastasis, while an increase uptake predicted progressive disease when evaluated at 7 weeks after radiotherapy, hormonal therapy, or chemotherapy for metastatic breast cancer
Jansson et al. (141)	16	[^11^C]methionine PET predicted response in 67% (8/12) of clinical responders as early as 6–13 days after the first course of chemotherapy.
Lindholm et al. (149)	13	[^11^C]methionine PET showed significant reduction in uptake (30–54%) in all six responding metastatic sites, whereas the decrease in uptake was lower in magnitude or showed an increase in stable or non-responding lesions, in metastatic breast cancer patients treated with polychemotherapy or hormones
**Bladder**		
Letocha et al. (148)	4	[^11^C]methionine PET identified patients who progressed after chemotherapy for localized or metastatic bladder cancer
Katz et al. (142)	1	In a patient with metastatic transitional cell carcinoma (TCC) unfit for platinum-based chemotherapy, being treated with Sunitinib, [^11^C]methionine PET showed a significantly decreased metabolic uptake in bone and lymph nodes 28 days after sunitinib initiation without any objective morphological changes, corroborated by objective tumor reduction on CT at 2 months after therapy initiation
**Choroidal melanoma**		
Tamura K (163)	1	[^11^C]methionine PET uptake when evaluated visually and semiquantitatively showed a significant decrease in tumor-to-brain ratio at ≥6 months after therapy and disappeared in 50% of the patients at 12 months after carbon-ion therapy
**Soft tissue sarcoma**		
Zhang et al. (170)		[^11^C]methionine PET was of prognostic value in patients with bone and soft tissue sarcoma treated by CIRT
Ghigi et al. (134)	9	The percentage variation in histological response (tumor grade regression) and SUVmax of [^18^F]FDG before and after neoadjuvant chemo-radiotherapy seems to discriminate between partial and complete response better than [^11^C]methionine
Rectal cancer		
Wieder et al. (168)	26	[^11^C]methionine PET aided tumor visualization, but the degree of reduction in [^11^C]methionine uptake post chemo-radiation did not correlate with the tumor response measured by pathologic evaluation. [^11^C]methionine PET may not be a good method for evaluating the response of radiotherapy in rectal cancer
Koizumi et al. (144)	53	[^11^C]methionine PET uptake decreased with CIRT but there were no significant correlations between imaging variables (SUV, tumor-to-normal tissue ratio) and other clinical parameters (distant metastasis and survival) in patients with rectal cancer
**Lung cancer**		
Kubota et al. (145)	21	A significant decrease in [^11^C]methionine uptake in responding human lung tumors 2 weeks after radiotherapy or chemotherapy, and the decrease preceded the shrinkage in tumor volume measured with CT
Ishimori et al. (139)	9	[^11^C]methionine PET did not provide additional information over FDG PET in lung cancer treated with stereotactic radiotherapy (SRT). Decline in [^11^C]methionine PET activity reflects acute reaction to SRT and the increase in activity in later time points denotes radiation-induced pneumonitis
**Lymphoma**		
Leskinen-Kallio et al. (147)	1	Demonstrated a decrease in [^11^C]methionine uptake with chemotherapy and radiotherapy in a patient with non-Hodgkin’s lymphoma (NHL)
Sawataishi et al. (160)	2	[^11^C]methionine PET improved lesion delineation compared to CT/MRI in PCNSL and predicted presence of residual tumors after radiotherapy in lesions involuting on CT
Ogawa et al. (156)	10	[^11^C]methionine PET is useful for the delineation of CNS lymphoma and for monitoring the therapeutic effect of irradiation. The extent of [^11^C]methionine accumulation in tumor tissue markedly decreased after radiation therapy
Tsuyuguchi et al. (166)	1	[^11^C]methionine PET is helpful in assessing the effect of chemotherapy earlier than is feasible with other methods in malignant scalp lymphoma

#### Leucine Analogs

Leucine is one of the preferential amino acid required for proliferating tumor cells and is, therefore, of interest in molecular imaging of anabolic cancer processes. 1-Amino-3-[^18^F]fluorocyclobutane-1-carboxylic acid (anti-[^18^F]FACBC), a synthetic non-natural leucine analog, has been widely studied in imaging brain (171, 172), prostate tumors (173, 174), and pulmonary lesions (175). The non-natural amino acids are not metabolized but are taken up through both the L-type transporter and the energy-dependent A-type transporter (176). The tracer accumulation in PCa cells correlates with the expression level of the alanine, serine, and cysteine preferring system-mediated amino acid transport with the large neutral amino acid transporter (LAT1) as an important transport system (177, 178). There are only two preclinical studies that evaluated the role of anti-[^18^F]FACBC in predicting response [Table 1; (121, 124)] and in these cases anti-[^18^F]FACBC PET provided useful information on early response. Future studies are eagerly awaited.

#### Tryptophan Analogs

Tryptophan is an essential amino acid required for biosynthesis of proteins, serotonin, and niacin in the brain and other tissues (179). The amino acid PET tracer alpha-[^11^C]methyl-L-tryptophan (AMT) is transported in tumor tissue by LAT1 but is not incorporated into proteins (180). Instead, AMT is utilized by the kynurenine immunomodulatory pathway (181). Under pathological conditions, induction of this pathway’s rate-limiting enzyme, indoleamine 2,3-dioxygenase (IDO), leads to increased metabolism of tryptophan and, thus, AMT accumulation (182). Tryptophan analogs have been widely studied in imaging high-grade gliomas (182, 183), CRPC (184), and neuroendocrine tumors (185). In a case report, Peng and co-workers suggested that AMT PET may be useful for assessing progression and therapeutic response of optic glioma (186). Further studies are eagerly awaited.

## Discussion

Several metabolic pathways are deranged in cancer in a proliferation-dependent or proliferation-independent manner. These metabolic pathways, particularly enhanced glycolysis, offer the opportunity to detect cancer often with high contrast. In this review article, we discuss about the role of established and evolving metabolism tracers for prediction/monitoring response to therapy. The effect of drug or radiation therapy on each metabolic phenotype ought to be carefully considered to enable assignment of biological and clinical relevance to the changes seen. Notably, these therapies may directly or indirectly inhibit tumor cell metabolism, or indeed the changes may simply reflect loss of cell viability and influence the timing of post-treatment scanning. For [^18^F]FDG PET, the effect of the so-called targeted or biologic therapies on response monitoring has been reviewed (26) with the suggestion that the drugs may directly affect GLUT/hexokinase expression or activity with changes occurring within hours to days after initiating treatment. This type of information is less mature when other metabolism tracers are considered. For example, as discussed above, only a few studies have attempted to directly link the biology of androgen deprivation to changes in the tumor labeled choline signal. Regarding imaging variables, different variables have been used in the assessment of non-FDG tracers (see Tables 1–4). Some of these variables, e.g., TMR, may be considered, for instance, when RT is the choice of therapy to account for the effect of radiation on normal tissues.

Whatever the mechanism of signal change, be it direct or via loss of cell viability, it is important to consider the intrinsic variability of the quantitative measure, as well as that magnitude of change (threshold) for response. For [^18^F]FDG uptake, the intrinsic measurement variability (without treatment) ranges from 10 to 20% in different tumor phenotypes (187, 188). Based on pooling together reproducibility data, a consensus for quantifying PET response by EORTC PET study group was reached (189). The tumor responses were graded as follows:

1) Complete metabolic response (CMR): complete resolution of FDG uptake.
2) Partial metabolic response (PMR): a decrease (across all lesions) of minimum of 15% in tumor SUV after one cycle or >25% after more than one cycle of chemotherapy.
3) Stable metabolic disease (SMD): an increase of <25% or a decrease of <15% in SUV, and no visible increase in extent of FDG tumor uptake (20% in longest dimension).
4) Progressive metabolic disease (PMD): an increase in FDG tumor SUV of >25% within tumor region defined on baseline scan; visible increase in extent of FDG tumor uptake (20% in longest dimension) or appearance of new FDG uptake in metastatic lesions.

More recently, PET Response Criteria in Solid Tumors (PERCIST) guidelines have been formulated (190). These are based on the premise that cancer response as assessed by PET is a continuous and time-dependent variable. The tumor responses were graded as follows:

1) CMR: visual disappearance of all metabolically active tumors.
2) PMR: more than a 30% decline and a 0.8-unit decline in SUL_peak_ between the most intense lesion before treatment and the most intense lesion after treatment, although not necessarily the same lesion.
3) SMD: not CMR, PMR, or PMD.
4) PMD: more than a 30% and 0.8-unit increase in SUL_peak_ or new lesions, if confirmed. A >75% increase in total lesion glycolysis is also proposed as another metric of progression.

The PERCIST criteria differ from the EORTC criteria in that the SUV is normalized to the lean body mass and five tumors (up to two per organ) with the most intense [^18^F]FDG uptake lesions being considered target lesions; SUL_mean_ is the recommended imaging variable, as it has better test–retest variability (8–10%), is statistically less susceptible to variance, and is less subjective due to clear definition of target lesions.

Notably, these criteria are specific for [^18^F]FDG PET and may differ for other tracers. For example, Kenny and co-workers have evaluated the reproducibility of [^11^C]choline in breast cancer (85). A decrease of 40% for SUV_30min_, and 24% for SUV_60min_, was classified statistically as response. However, it is not clear if these criteria could be widely applied across different tumor sites or across different PET tracers, as the intrinsic variability may be isotope, patient, or scanner related.

In the future, further evaluation is required to assess the role of metabolic-PET imaging in assessing response to treatment and follow-up after treatment. These include what the optimal time (early or delayed) for performing the scan after treatment is, what the relevant imaging variables for predicting response are, how often to scan, whether imaging sensitivity and specificity are sufficient to predict response or progression, and whether changes in imaging variables can be used as surrogates for predicting patient outcomes. Future studies will need to be designed to establish the answers to these questions.

## Conclusion

In this article, we aimed to give an overview of metabolic processes imaged by PET and focused on both established and evolving radioprobes to detect tumor glycolysis, choline metabolism, intracellular transport of glutamine, and other amino acids, as well as fatty acid metabolism. In particular, we emphasize the role of radiolabeled choline, acetate, and amino acid tracers for monitoring efficacy or predicting response to new therapies that directly or indirectly inhibit tumor cell metabolism. The optimal imaging time point, pertinent imaging variable, and criteria for response will require further interrogation.

## Author Contributions

AC and EA have contributed to the conception, layout of the review, and reviewed and proof read.

## Conflict of Interest Statement

The authors declare that the research was conducted in the absence of any commercial or financial relationships that could be construed as a potential conflict of interest.
